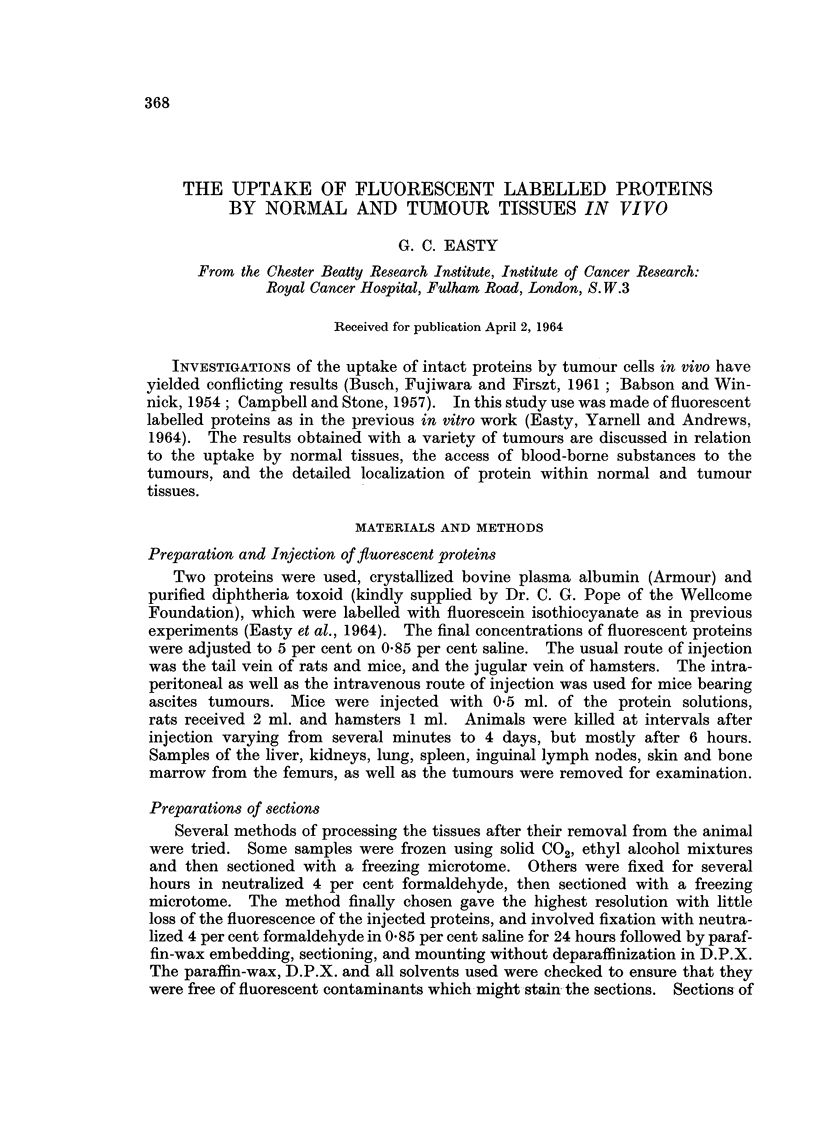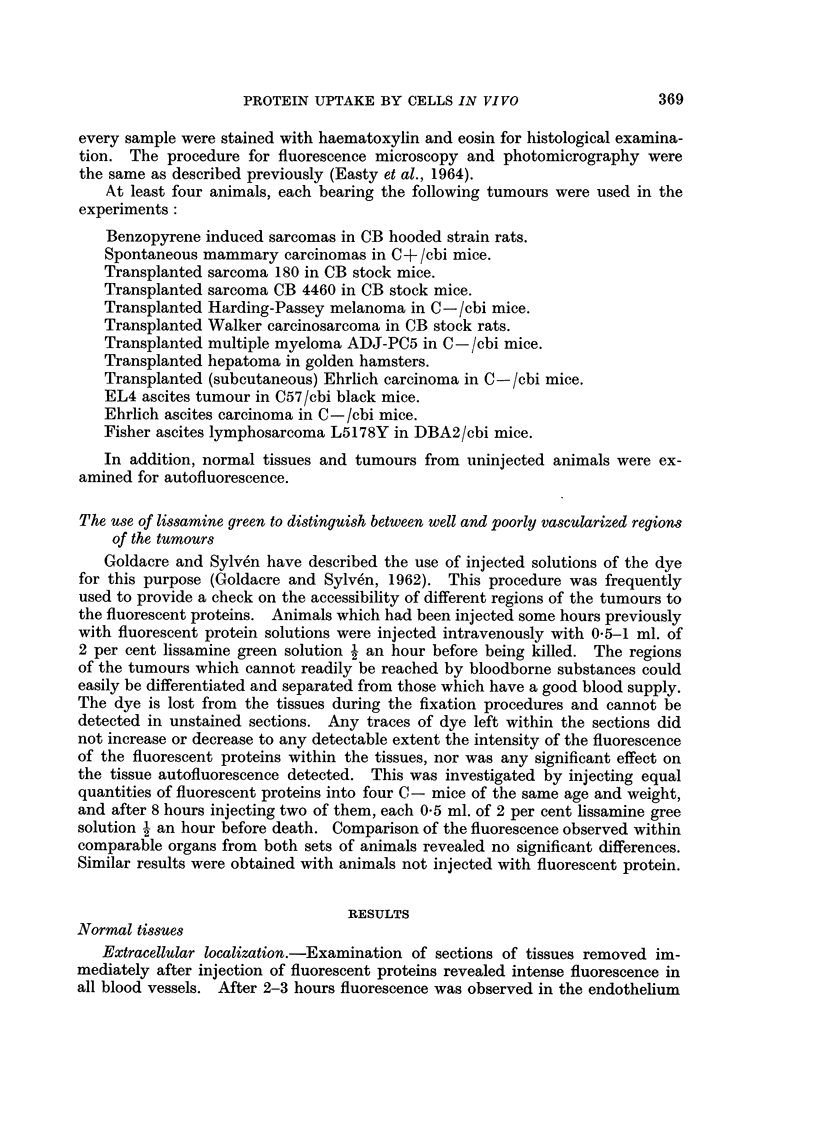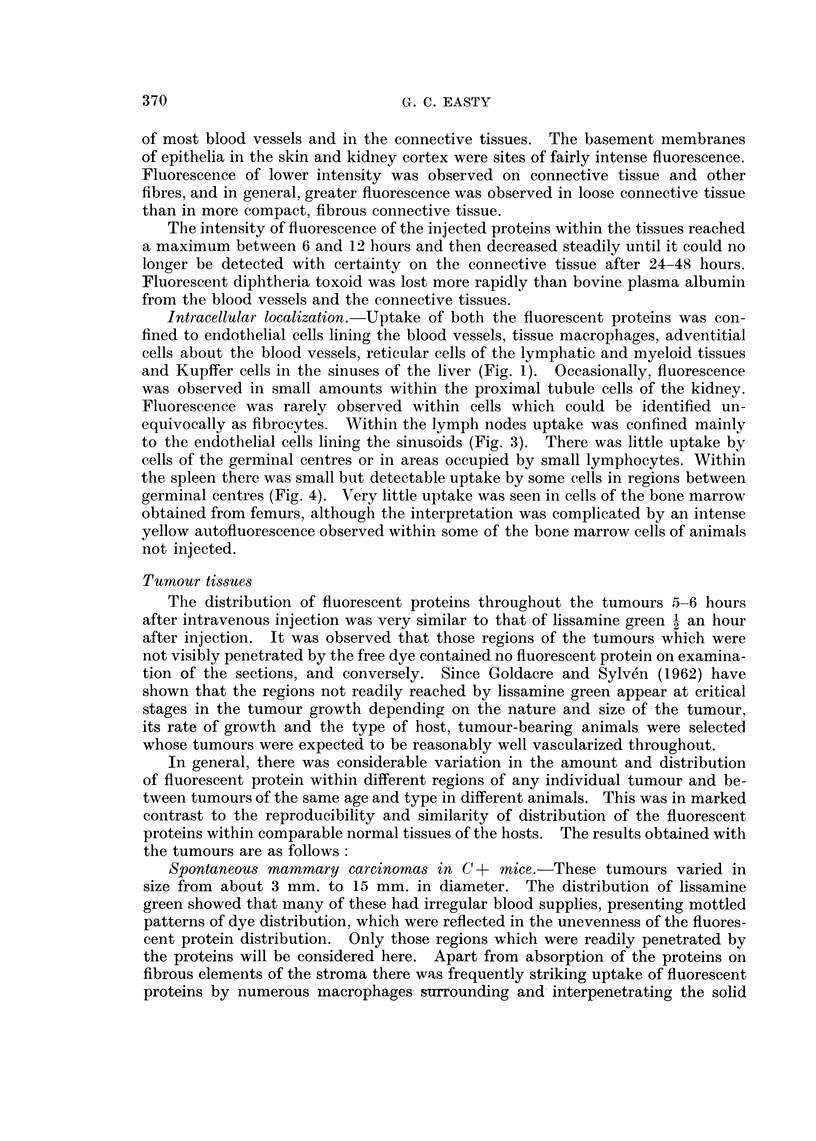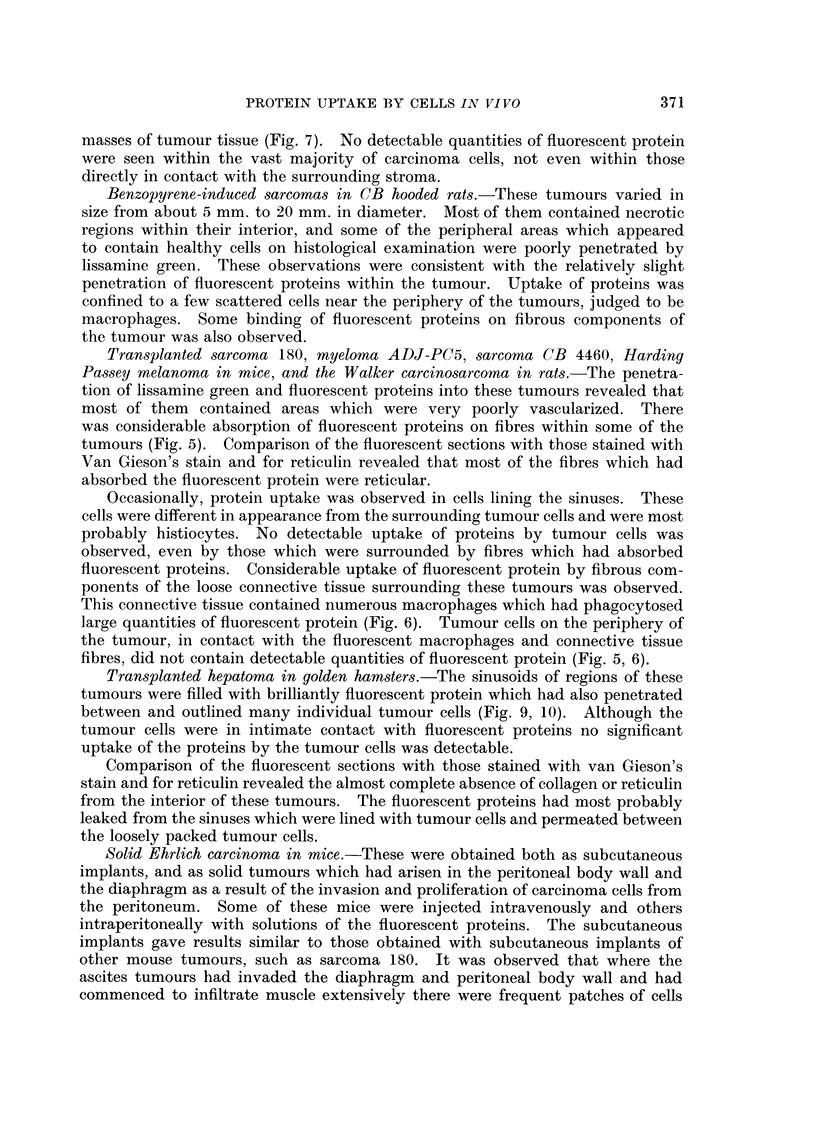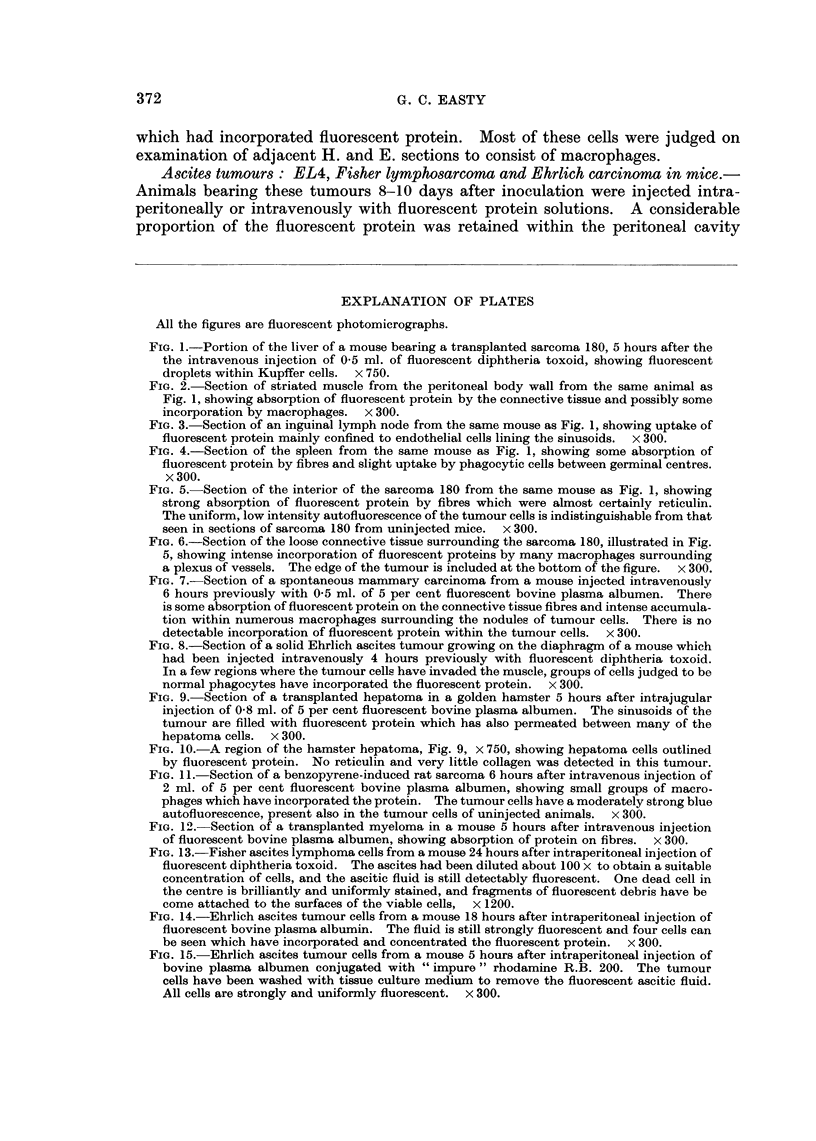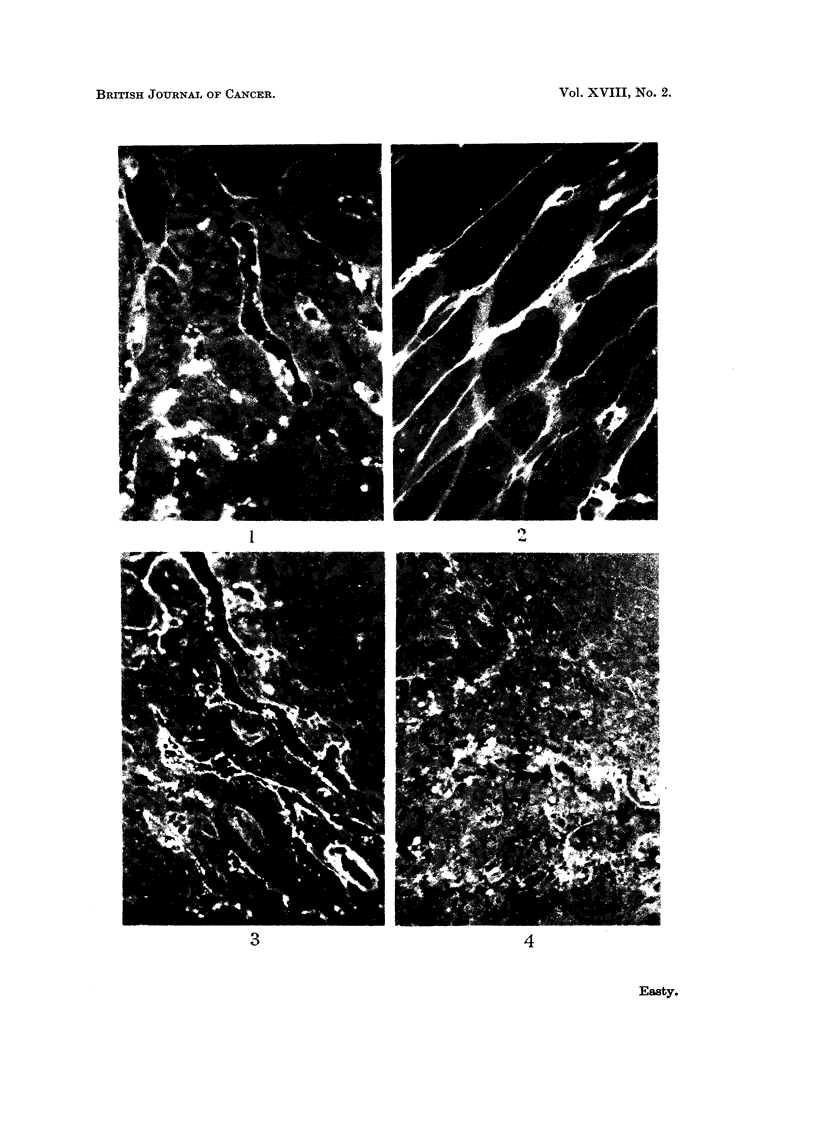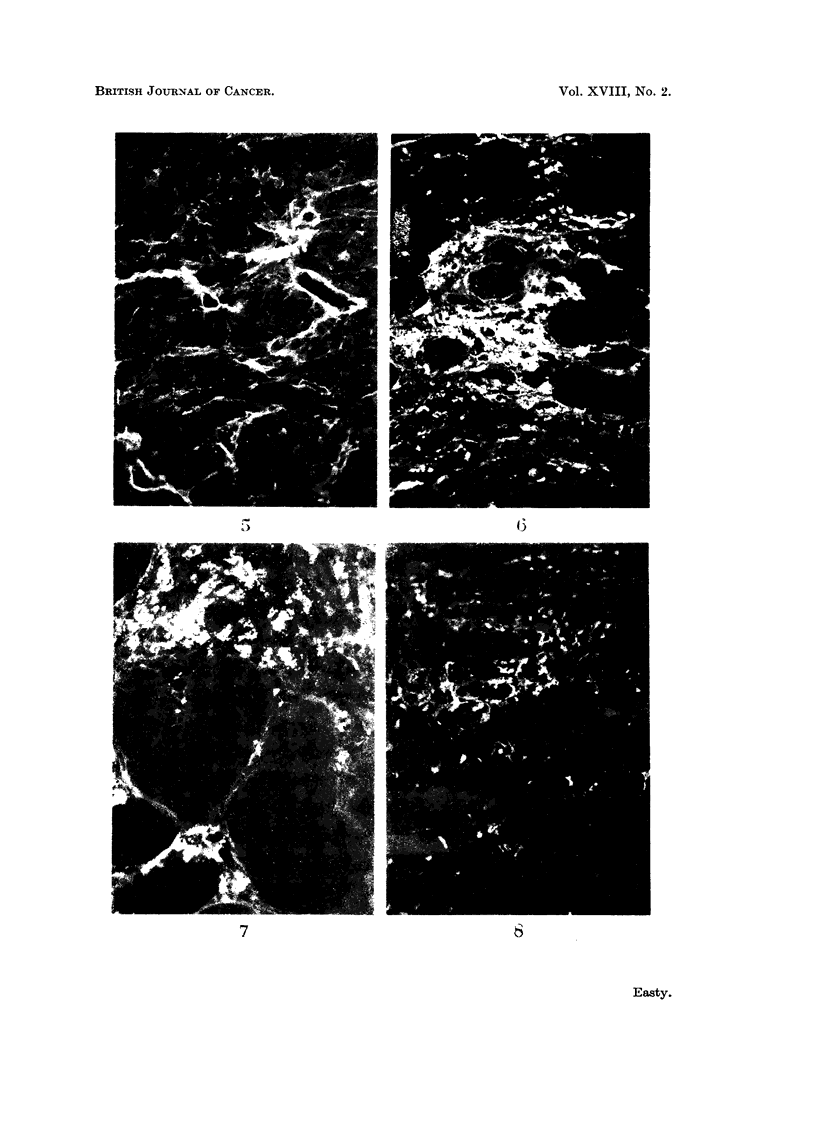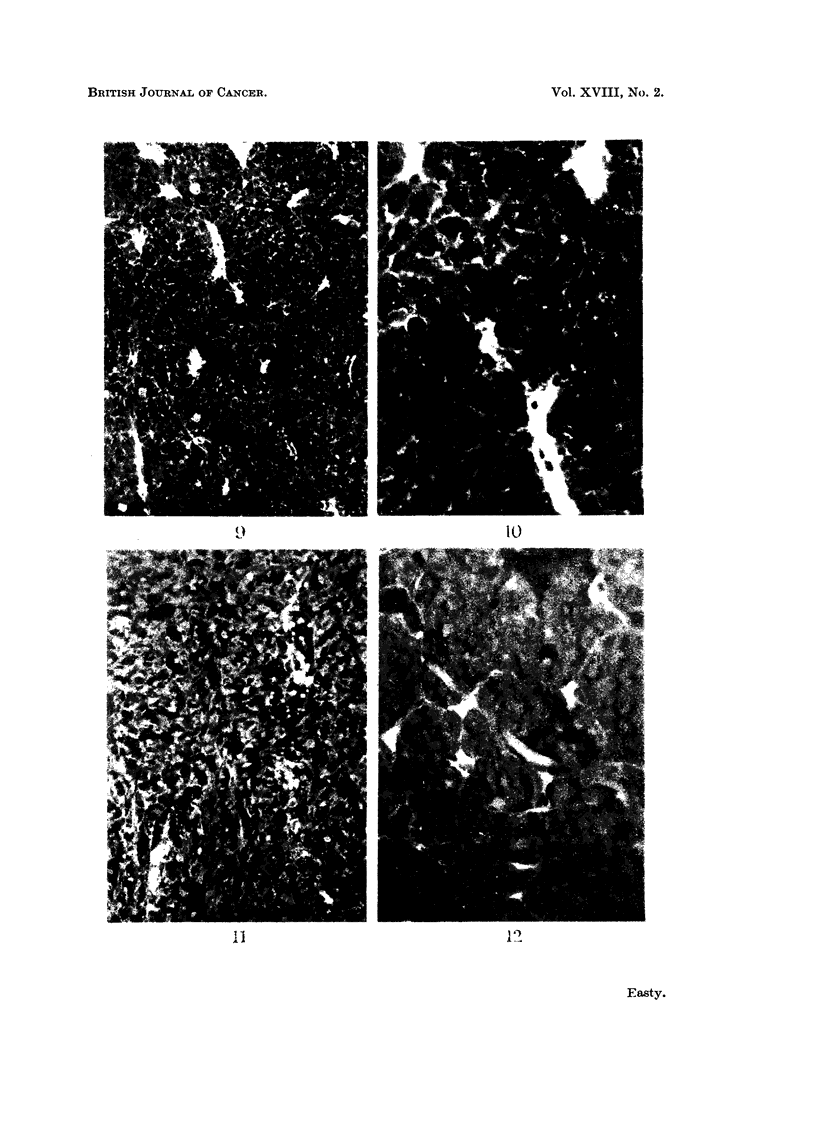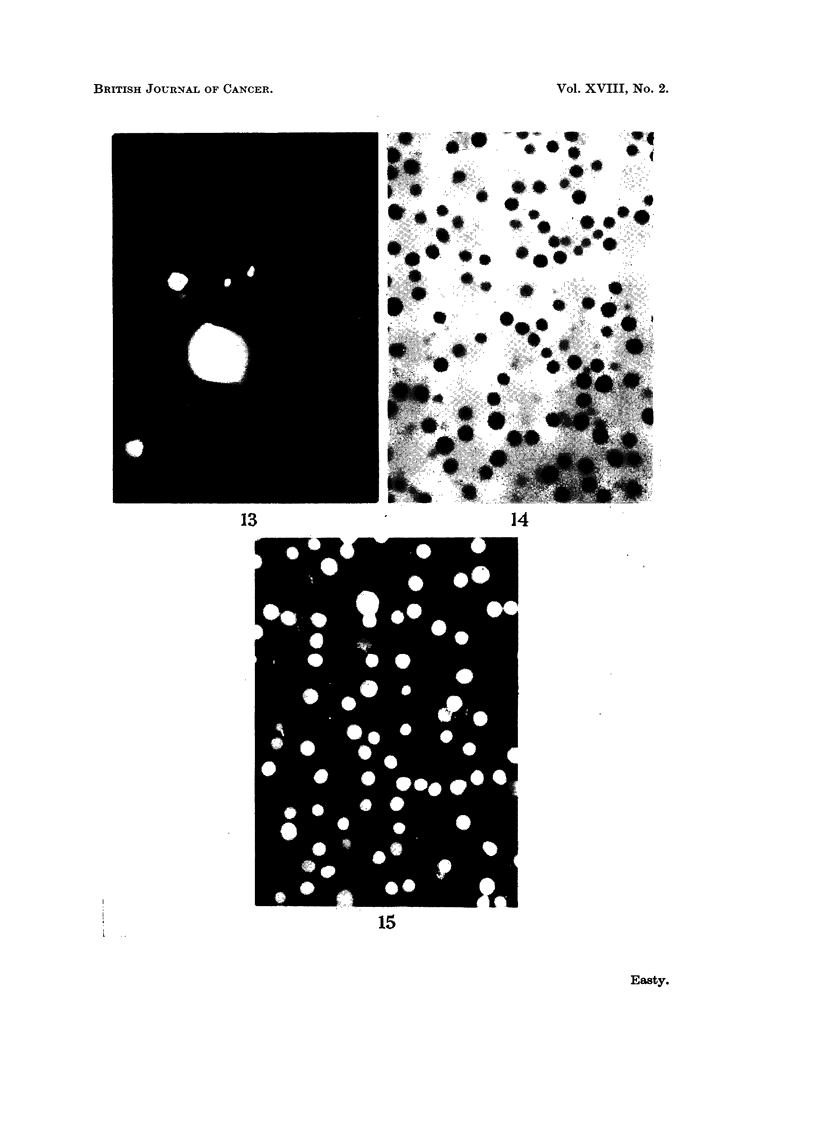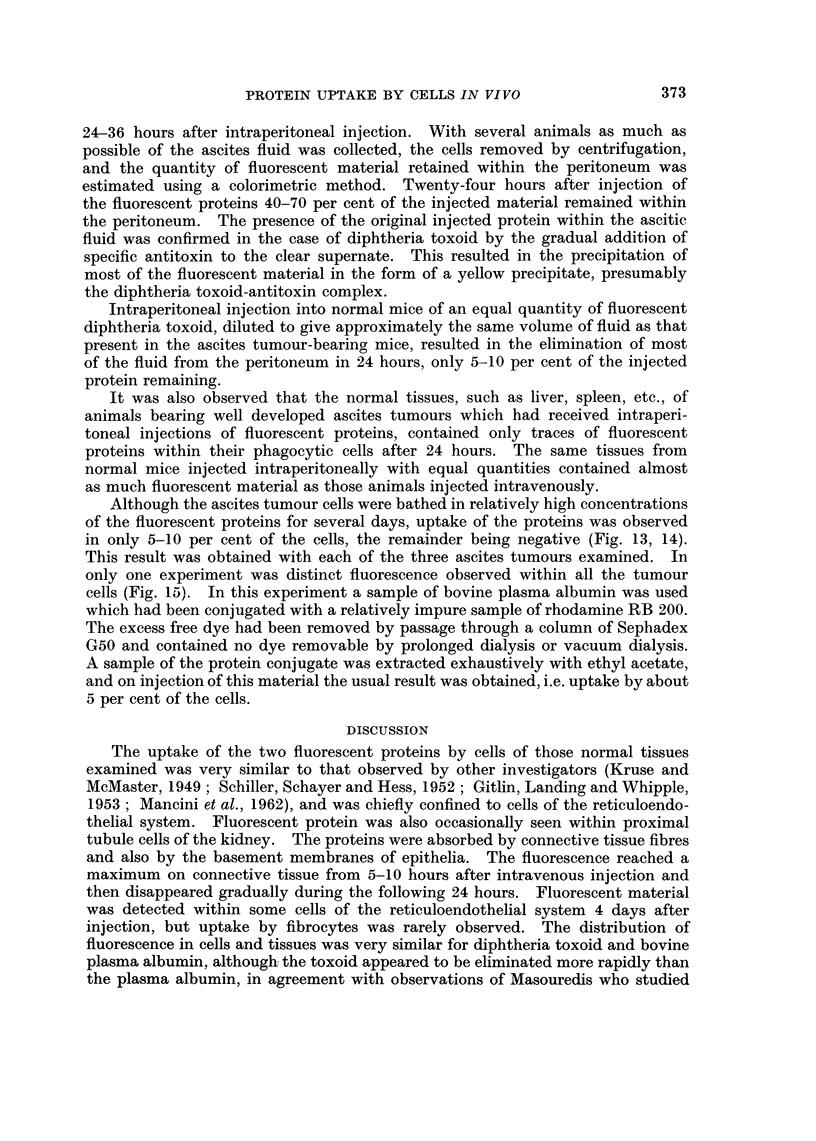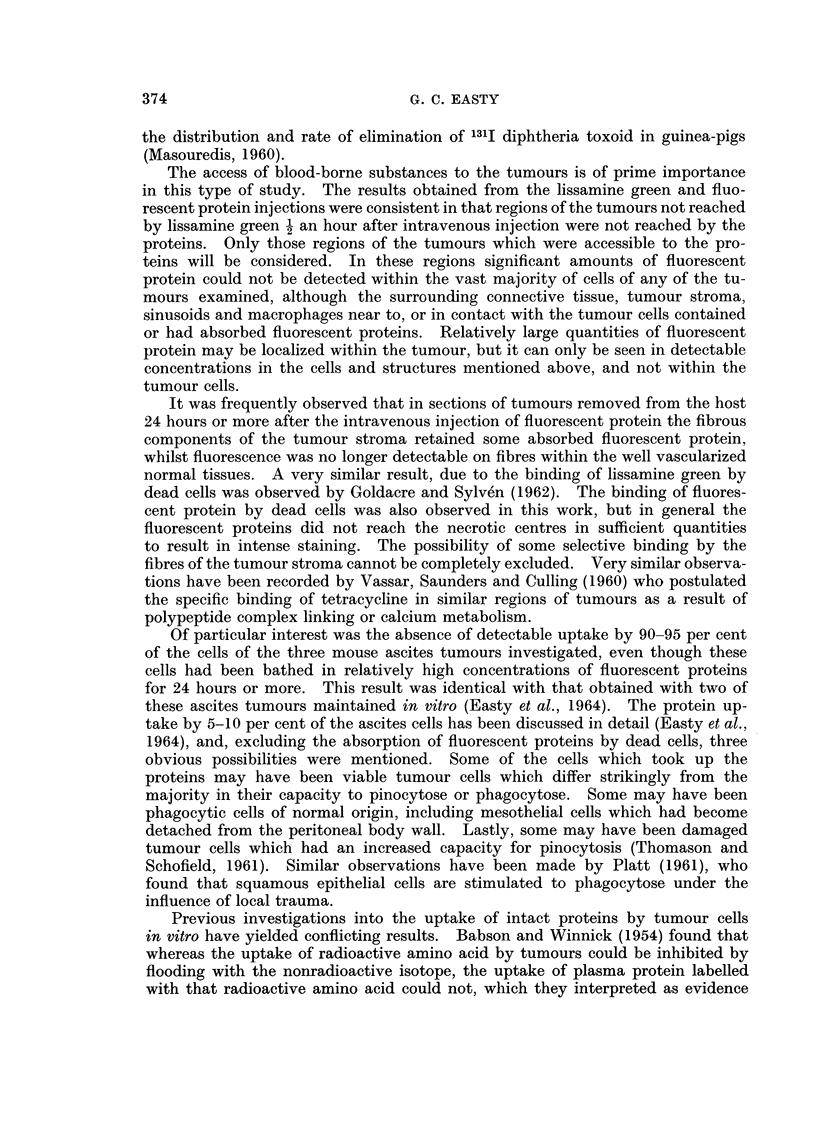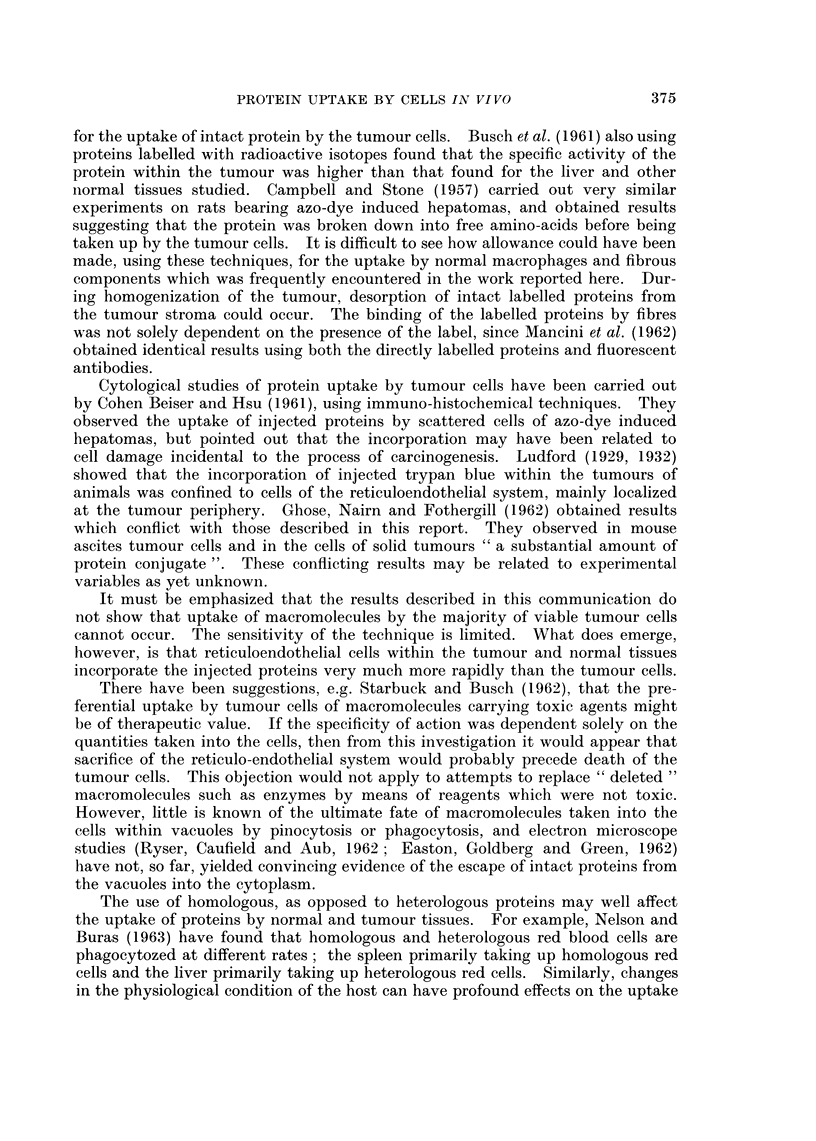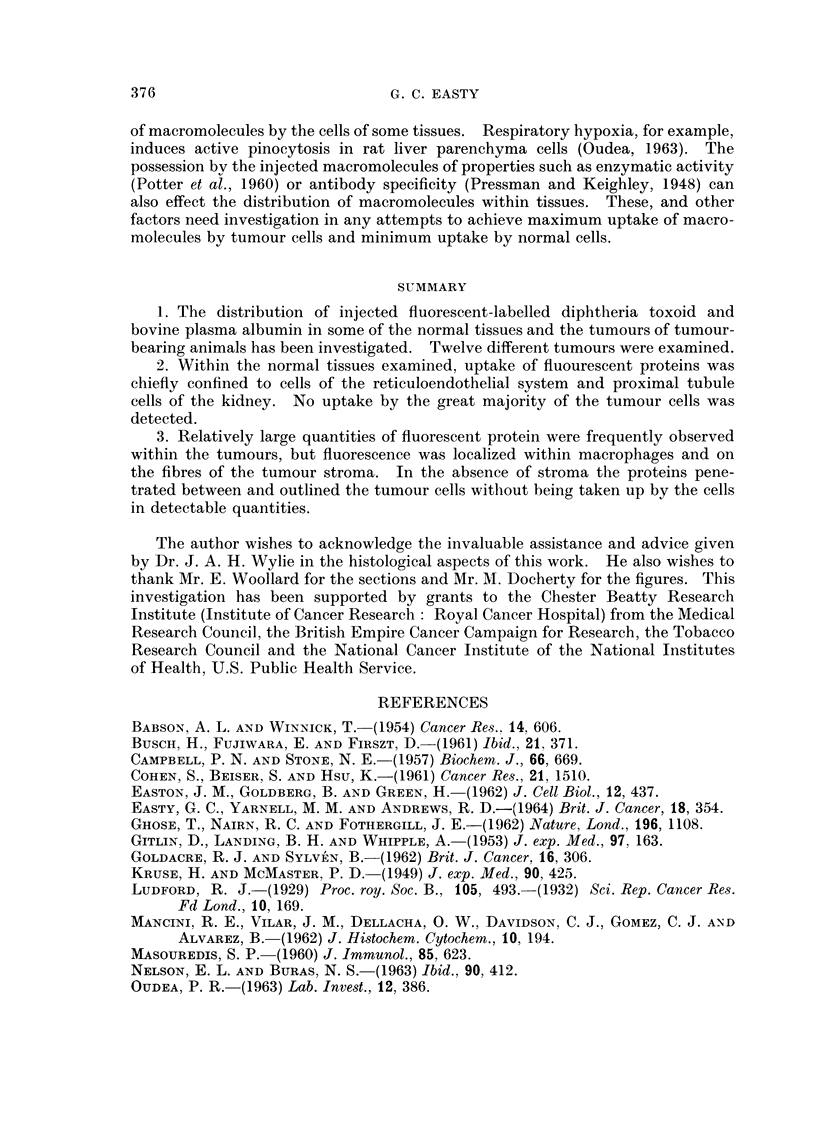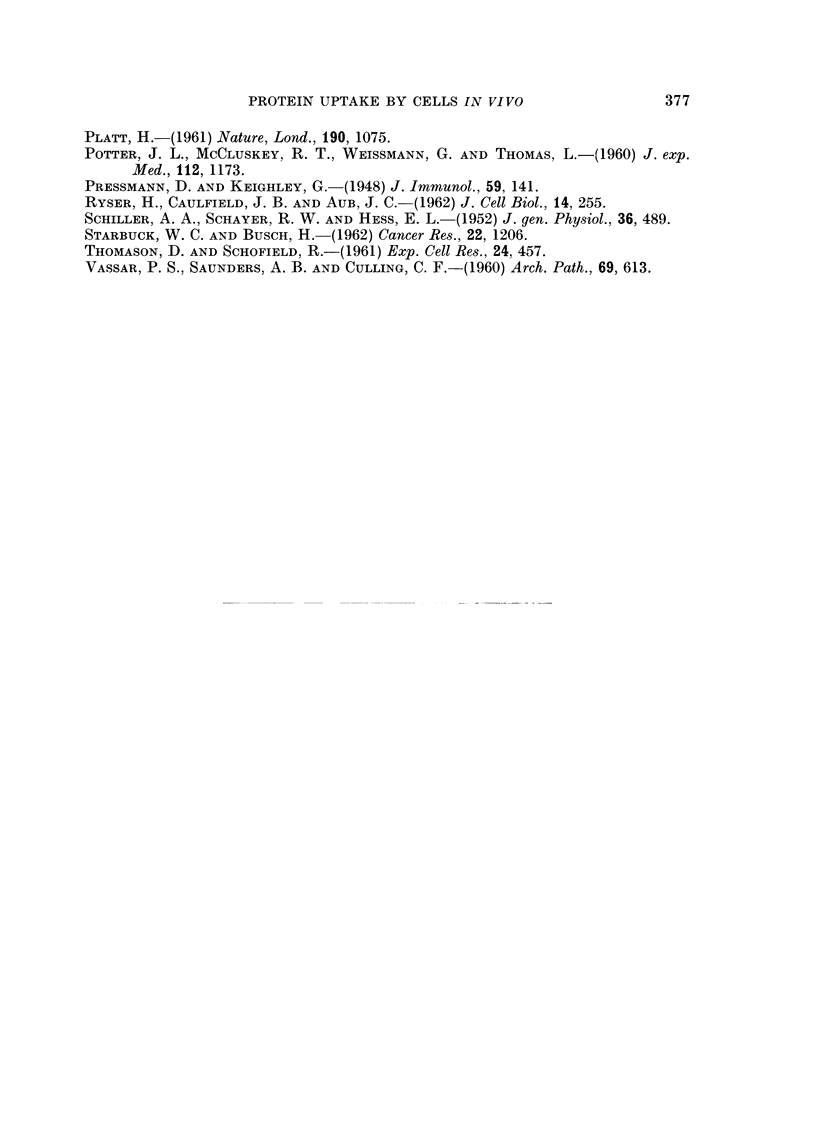# The Uptake of Fluorescent Labelled Proteins by Normal and Tumour Tissues In Vivo

**DOI:** 10.1038/bjc.1964.42

**Published:** 1964-06

**Authors:** G. C. Easty

## Abstract

**Images:**


### THE UPTAKE OF FLUORESCENT LABELLED PROTEINS BY NORMAL AND TUMOUR TISSUES *IN VIVO*

G. C. EASTY

*From the Chester Beatty Research Institute, Institute of Cancer Research: Royal Cancer Hospital, Fulham Road, London, S.W.3*

Received for publication April 2, 1964

INVESTIGATIONS of the uptake of intact proteins by tumour cells *in vivo* have yielded conflicting results (Busch, Fujiwara and Firszt, 1961 ; Babson and Winnick, 1954 ; Campbell and Stone, 1957). In this study use was made of fluorescent labelled proteins as in the previous *in vitro* work (Easty, Yarnell and Andrews, 1964). The results obtained with a variety of tumours are discussed in relation to the uptake by normal tissues, the access of blood-borne substances to the tumours, and the detailed localization of protein within normal and tumour tissues.

### MATERIALS AND METHODS

### *Preparation and Injection of fluorescent proteins*

Two proteins were used, crystallized bovine plasma albumin (Armour) and purified diphtheria toxoid (kindly supplied by Dr. C. G. Pope of the Wellcome Foundation), which were labelled with fluorescein isothiocyanate as in previous experiments (Easty *et al.*, 1964). The final concentrations of fluorescent proteins were adjusted to 5 per cent on 0·85 per cent saline. The usual route of injection was the tail vein of rats and mice, and the jugular vein of hamsters. The intraperitoneal as well as the intravenous route of injection was used for mice bearing ascites tumours. Mice were injected with 0·5 ml. of the protein solutions, rats received 2 ml. and hamsters 1 ml. Animals were killed at intervals after injection varying from several minutes to 4 days, but mostly after 6 hours. Samples of the liver, kidneys, lung, spleen, inguinal lymph nodes, skin and bone marrow from the femurs, as well as the tumours were removed for examination.

### *Preparations of sections*

Several methods of processing the tissues after their removal from the animal were tried. Some samples were frozen using solid $CO_2$, ethyl alcohol mixtures and then sectioned with a freezing microtome. Others were fixed for several hours in neutralized 4 per cent formaldehyde, then sectioned with a freezing microtome. The method finally chosen gave the highest resolution with little loss of the fluorescence of the injected proteins, and involved fixation with neutralized 4 per cent formaldehyde in 0·85 per cent saline for 24 hours followed by paraffin-wax embedding, sectioning, and mounting without deparaffinization in D.P.X. The paraffin-wax, D.P.X. and all solvents used were checked to ensure that they were free of fluorescent contaminants which might stain the sections. Sections of every sample were stained with haematoxylin and eosin for histological examination. The procedure for fluorescence microscopy and photomicrography were the same as described previously (Easty *et al.*, 1964).

At least four animals, each bearing the following tumours were used in the experiments :

Benzopyrene induced sarcomas in CB hooded strain rats.
Spontaneous mammary carcinomas in C+/cbi mice.
Transplanted sarcoma 180 in CB stock mice.
Transplanted sarcoma CB 4460 in CB stock mice.
Transplanted Harding-Passey melanoma in C—/cbi mice.
Transplanted Walker carcinosarcoma in CB stock rats.
Transplanted multiple myeloma ADJ-PC5 in C—/cbi mice.
Transplanted hepatoma in golden hamsters.
Transplanted (subcutaneous) Ehrlich carcinoma in C—/cbi mice.
EL4 ascites tumour in C57/cbi black mice.
Ehrlich ascites carcinoma in C—/cbi mice.
Fisher ascites lymphosarcoma L5178Y in DBA2/cbi mice.

In addition, normal tissues and tumours from uninjected animals were examined for autofluorescence.

*The use of lissamine green to distinguish between well and poorly vascularized regions of the tumours*

Goldacre and Sylvén have described the use of injected solutions of the dye for this purpose (Goldacre and Sylvén, 1962). This procedure was frequently used to provide a check on the accessibility of different regions of the tumours to the fluorescent proteins. Animals which had been injected some hours previously with fluorescent protein solutions were injected intravenously with 0·5–1 ml. of 2 per cent lissamine green solution ½ an hour before being killed. The regions of the tumours which cannot readily be reached by bloodborne substances could easily be differentiated and separated from those which have a good blood supply. The dye is lost from the tissues during the fixation procedures and cannot be detected in unstained sections. Any traces of dye left within the sections did not increase or decrease to any detectable extent the intensity of the fluorescence of the fluorescent proteins within the tissues, nor was any significant effect on the tissue autofluorescence detected. This was investigated by injecting equal quantities of fluorescent proteins into four C— mice of the same age and weight, and after 8 hours injecting two of them, each 0·5 ml. of 2 per cent lissamine gree solution ½ an hour before death. Comparison of the fluorescence observed within comparable organs from both sets of animals revealed no significant differences. Similar results were obtained with animals not injected with fluorescent protein.

### RESULTS

*Normal tissues*

*Extracellular localization.*—Examination of sections of tissues removed immediately after injection of fluorescent proteins revealed intense fluorescence in all blood vessels. After 2–3 hours fluorescence was observed in the endothelium of most blood vessels and in the connective tissues. The basement membranes of epithelia in the skin and kidney cortex were sites of fairly intense fluorescence. Fluorescence of lower intensity was observed on connective tissue and other fibres, and in general, greater fluorescence was observed in loose connective tissue than in more compact, fibrous connective tissue.

The intensity of fluorescence of the injected proteins within the tissues reached a maximum between 6 and 12 hours and then decreased steadily until it could no longer be detected with certainty on the connective tissue after 24–48 hours. Fluorescent diphtheria toxoid was lost more rapidly than bovine plasma albumin from the blood vessels and the connective tissues.

*Intracellular localization.*—Uptake of both the fluorescent proteins was confined to endothelial cells lining the blood vessels, tissue macrophages, adventitial cells about the blood vessels, reticular cells of the lymphatic and myeloid tissues and Kupffer cells in the sinuses of the liver (Fig. 1). Occasionally, fluorescence was observed in small amounts within the proximal tubule cells of the kidney. Fluorescence was rarely observed within cells which could be identified unequivocally as fibrocytes. Within the lymph nodes uptake was confined mainly to the endothelial cells lining the sinusoids (Fig. 3). There was little uptake by cells of the germinal centres or in areas occupied by small lymphocytes. Within the spleen there was small but detectable uptake by some cells in regions between germinal centres (Fig. 4). Very little uptake was seen in cells of the bone marrow obtained from femurs, although the interpretation was complicated by an intense yellow autofluorescence observed within some of the bone marrow cells of animals not injected.

*Tumour tissues*

The distribution of fluorescent proteins throughout the tumours 5–6 hours after intravenous injection was very similar to that of lissamine green $\frac{1}{2}$ an hour after injection. It was observed that those regions of the tumours which were not visibly penetrated by the free dye contained no fluorescent protein on examination of the sections, and conversely. Since Goldacre and Sylvén (1962) have shown that the regions not readily reached by lissamine green appear at critical stages in the tumour growth depending on the nature and size of the tumour, its rate of growth and the type of host, tumour-bearing animals were selected whose tumours were expected to be reasonably well vascularized throughout.

In general, there was considerable variation in the amount and distribution of fluorescent protein within different regions of any individual tumour and between tumours of the same age and type in different animals. This was in marked contrast to the reproducibility and similarity of distribution of the fluorescent proteins within comparable normal tissues of the hosts. The results obtained with the tumours are as follows :

*Spontaneous mammary carcinomas in C+ mice.*—These tumours varied in size from about 3 mm. to 15 mm. in diameter. The distribution of lissamine green showed that many of these had irregular blood supplies, presenting mottled patterns of dye distribution, which were reflected in the unevenness of the fluorescent protein distribution. Only those regions which were readily penetrated by the proteins will be considered here. Apart from absorption of the proteins on fibrous elements of the stroma there was frequently striking uptake of fluorescent proteins by numerous macrophages surrounding and interpenetrating the solid masses of tumour tissue (Fig. 7). No detectable quantities of fluorescent protein were seen within the vast majority of carcinoma cells, not even within those directly in contact with the surrounding stroma.

*Benzopyrene-induced sarcomas in CB hooded rats.*—These tumours varied in size from about 5 mm. to 20 mm. in diameter. Most of them contained necrotic regions within their interior, and some of the peripheral areas which appeared to contain healthy cells on histological examination were poorly penetrated by lissamine green. These observations were consistent with the relatively slight penetration of fluorescent proteins within the tumour. Uptake of proteins was confined to a few scattered cells near the periphery of the tumours, judged to be macrophages. Some binding of fluorescent proteins on fibrous components of the tumour was also observed.

*Transplanted sarcoma* 180, *myeloma ADJ-PC*5, *sarcoma CB* 4460, *Harding Passey melanoma in mice, and the Walker carcinosarcoma in rats.*—The penetration of lissamine green and fluorescent proteins into these tumours revealed that most of them contained areas which were very poorly vascularized. There was considerable absorption of fluorescent proteins on fibres within some of the tumours (Fig. 5). Comparison of the fluorescent sections with those stained with Van Gieson's stain and for reticulin revealed that most of the fibres which had absorbed the fluorescent protein were reticular.

Occasionally, protein uptake was observed in cells lining the sinuses. These cells were different in appearance from the surrounding tumour cells and were most probably histiocytes. No detectable uptake of proteins by tumour cells was observed, even by those which were surrounded by fibres which had absorbed fluorescent proteins. Considerable uptake of fluorescent protein by fibrous components of the loose connective tissue surrounding these tumours was observed. This connective tissue contained numerous macrophages which had phagocytosed large quantities of fluorescent protein (Fig. 6). Tumour cells on the periphery of the tumour, in contact with the fluorescent macrophages and connective tissue fibres, did not contain detectable quantities of fluorescent protein (Fig. 5, 6).

*Transplanted hepatoma in golden hamsters.*—The sinusoids of regions of these tumours were filled with brilliantly fluorescent protein which had also penetrated between and outlined many individual tumour cells (Fig. 9, 10). Although the tumour cells were in intimate contact with fluorescent proteins no significant uptake of the proteins by the tumour cells was detectable.

Comparison of the fluorescent sections with those stained with van Gieson's stain and for reticulin revealed the almost complete absence of collagen or reticulin from the interior of these tumours. The fluorescent proteins had most probably leaked from the sinuses which were lined with tumour cells and permeated between the loosely packed tumour cells.

*Solid Ehrlich carcinoma in mice.*—These were obtained both as subcutaneous implants, and as solid tumours which had arisen in the peritoneal body wall and the diaphragm as a result of the invasion and proliferation of carcinoma cells from the peritoneum. Some of these mice were injected intravenously and others intraperitoneally with solutions of the fluorescent proteins. The subcutaneous implants gave results similar to those obtained with subcutaneous implants of other mouse tumours, such as sarcoma 180. It was observed that where the ascites tumours had invaded the diaphragm and peritoneal body wall and had commenced to infiltrate muscle extensively there were frequent patches of cells which had incorporated fluorescent protein. Most of these cells were judged on examination of adjacent H. and E. sections to consist of macrophages.

*Ascites tumours : EL4, Fisher lymphosarcoma and Ehrlich carcinoma in mice.*—Animals bearing these tumours 8–10 days after inoculation were injected intraperitoneally or intravenously with fluorescent protein solutions. A considerable proportion of the fluorescent protein was retained within the peritoneal cavity

---

### EXPLANATION OF PLATES

All the figures are fluorescent photomicrographs.

Fig. 1.—Portion of the liver of a mouse bearing a transplanted sarcoma 180, 5 hours after the the intravenous injection of 0·5 ml. of fluorescent diphtheria toxoid, showing fluorescent droplets within Kupffer cells. ×750.

Fig. 2.—Section of striated muscle from the peritoneal body wall from the same animal as Fig. 1, showing absorption of fluorescent protein by the connective tissue and possibly some incorporation by macrophages. ×300.

Fig. 3.—Section of an inguinal lymph node from the same mouse as Fig. 1, showing uptake of fluorescent protein mainly confined to endothelial cells lining the sinusoids. ×300.

Fig. 4.—Section of the spleen from the same mouse as Fig. 1, showing some absorption of fluorescent protein by fibres and slight uptake by phagocytic cells between germinal centres. ×300.

Fig. 5.—Section of the interior of the sarcoma 180 from the same mouse as Fig. 1, showing strong absorption of fluorescent protein by fibres which were almost certainly reticulin. The uniform, low intensity autofluorescence of the tumour cells is indistinguishable from that seen in sections of sarcoma 180 from uninjected mice. ×300.

Fig. 6.—Section of the loose connective tissue surrounding the sarcoma 180, illustrated in Fig. 5, showing intense incorporation of fluorescent proteins by many macrophages surrounding a plexus of vessels. The edge of the tumour is included at the bottom of the figure. ×300.

Fig. 7.—Section of a spontaneous mammary carcinoma from a mouse injected intravenously 6 hours previously with 0·5 ml. of 5 per cent fluorescent bovine plasma albumen. There is some absorption of fluorescent protein on the connective tissue fibres and intense accumulation within numerous macrophages surrounding the nodules of tumour cells. There is no detectable incorporation of fluorescent protein within the tumour cells. ×300.

Fig. 8.—Section of a solid Ehrlich ascites tumour growing on the diaphragm of a mouse which had been injected intravenously 4 hours previously with fluorescent diphtheria toxoid. In a few regions where the tumour cells have invaded the muscle, groups of cells judged to be normal phagocytes have incorporated the fluorescent protein. ×300.

Fig. 9.—Section of a transplanted hepatoma in a golden hamster 5 hours after intrajugular injection of 0·8 ml. of 5 per cent fluorescent bovine plasma albumen. The sinusoids of the tumour are filled with fluorescent protein which has also permeated between many of the hepatoma cells. ×300.

Fig. 10.—A region of the hamster hepatoma, Fig. 9, ×750, showing hepatoma cells outlined by fluorescent protein. No reticulin and very little collagen was detected in this tumour.

Fig. 11.—Section of a benzopyrene-induced rat sarcoma 6 hours after intravenous injection of 2 ml. of 5 per cent fluorescent bovine plasma albumen, showing small groups of macrophages which have incorporated the protein. The tumour cells have a moderately strong blue autofluorescence, present also in the tumour cells of uninjected animals. ×300.

Fig. 12.—Section of a transplanted myeloma in a mouse 5 hours after intravenous injection of fluorescent bovine plasma albumen, showing absorption of protein on fibres. ×300.

Fig. 13.—Fisher ascites lymphoma cells from a mouse 24 hours after intraperitoneal injection of fluorescent diphtheria toxoid. The ascites had been diluted about 100× to obtain a suitable concentration of cells, and the ascitic fluid is still detectably fluorescent. One dead cell in the centre is brilliantly and uniformly stained, and fragments of fluorescent debris have be come attached to the surfaces of the viable cells, ×1200.

Fig. 14.—Ehrlich ascites tumour cells from a mouse 18 hours after intraperitoneal injection of fluorescent bovine plasma albumin. The fluid is still strongly fluorescent and four cells can be seen which have incorporated and concentrated the fluorescent protein. ×300.

Fig. 15.—Ehrlich ascites tumour cells from a mouse 5 hours after intraperitoneal injection of bovine plasma albumen conjugated with " impure " rhodamine R.B. 200. The tumour cells have been washed with tissue culture medium to remove the fluorescent ascitic fluid. All cells are strongly and uniformly fluorescent. ×300.

1

2

3

4

Easty.

5

6

7

8

Easty.

9

10

11

12

Easty.

13

14

15

Easty.

24–36 hours after intraperitoneal injection. With several animals as much as possible of the ascites fluid was collected, the cells removed by centrifugation, and the quantity of fluorescent material retained within the peritoneum was estimated using a colorimetric method. Twenty-four hours after injection of the fluorescent proteins 40–70 per cent of the injected material remained within the peritoneum. The presence of the original injected protein within the ascitic fluid was confirmed in the case of diphtheria toxoid by the gradual addition of specific antitoxin to the clear supernate. This resulted in the precipitation of most of the fluorescent material in the form of a yellow precipitate, presumably the diphtheria toxoid-antitoxin complex.

Intraperitoneal injection into normal mice of an equal quantity of fluorescent diphtheria toxoid, diluted to give approximately the same volume of fluid as that present in the ascites tumour-bearing mice, resulted in the elimination of most of the fluid from the peritoneum in 24 hours, only 5–10 per cent of the injected protein remaining.

It was also observed that the normal tissues, such as liver, spleen, etc., of animals bearing well developed ascites tumours which had received intraperitoneal injections of fluorescent proteins, contained only traces of fluorescent proteins within their phagocytic cells after 24 hours. The same tissues from normal mice injected intraperitoneally with equal quantities contained almost as much fluorescent material as those animals injected intravenously.

Although the ascites tumour cells were bathed in relatively high concentrations of the fluorescent proteins for several days, uptake of the proteins was observed in only 5–10 per cent of the cells, the remainder being negative (Fig. 13, 14). This result was obtained with each of the three ascites tumours examined. In only one experiment was distinct fluorescence observed within all the tumour cells (Fig. 15). In this experiment a sample of bovine plasma albumin was used which had been conjugated with a relatively impure sample of rhodamine RB 200. The excess free dye had been removed by passage through a column of Sephadex G50 and contained no dye removable by prolonged dialysis or vacuum dialysis. A sample of the protein conjugate was extracted exhaustively with ethyl acetate, and on injection of this material the usual result was obtained, i.e. uptake by about 5 per cent of the cells.

### DISCUSSION

The uptake of the two fluorescent proteins by cells of those normal tissues examined was very similar to that observed by other investigators (Kruse and McMaster, 1949 ; Schiller, Schayer and Hess, 1952 ; Gitlin, Landing and Whipple, 1953 ; Mancini *et al.*, 1962), and was chiefly confined to cells of the reticuloendothelial system. Fluorescent protein was also occasionally seen within proximal tubule cells of the kidney. The proteins were absorbed by connective tissue fibres and also by the basement membranes of epithelia. The fluorescence reached a maximum on connective tissue from 5–10 hours after intravenous injection and then disappeared gradually during the following 24 hours. Fluorescent material was detected within some cells of the reticuloendothelial system 4 days after injection, but uptake by fibrocytes was rarely observed. The distribution of fluorescence in cells and tissues was very similar for diphtheria toxoid and bovine plasma albumin, although the toxoid appeared to be eliminated more rapidly than the plasma albumin, in agreement with observations of Masouredis who studied the distribution and rate of elimination of $^{131}I$ diphtheria toxoid in guinea-pigs (Masouredis, 1960).

The access of blood-borne substances to the tumours is of prime importance in this type of study. The results obtained from the lissamine green and fluorescent protein injections were consistent in that regions of the tumours not reached by lissamine green $\frac{1}{2}$ an hour after intravenous injection were not reached by the proteins. Only those regions of the tumours which were accessible to the proteins will be considered. In these regions significant amounts of fluorescent protein could not be detected within the vast majority of cells of any of the tumours examined, although the surrounding connective tissue, tumour stroma, sinusoids and macrophages near to, or in contact with the tumour cells contained or had absorbed fluorescent proteins. Relatively large quantities of fluorescent protein may be localized within the tumour, but it can only be seen in detectable concentrations in the cells and structures mentioned above, and not within the tumour cells.

It was frequently observed that in sections of tumours removed from the host 24 hours or more after the intravenous injection of fluorescent protein the fibrous components of the tumour stroma retained some absorbed fluorescent protein, whilst fluorescence was no longer detectable on fibres within the well vascularized normal tissues. A very similar result, due to the binding of lissamine green by dead cells was observed by Goldacre and Sylvén (1962). The binding of fluorescent protein by dead cells was also observed in this work, but in general the fluorescent proteins did not reach the necrotic centres in sufficient quantities to result in intense staining. The possibility of some selective binding by the fibres of the tumour stroma cannot be completely excluded. Very similar observations have been recorded by Vassar, Saunders and Culling (1960) who postulated the specific binding of tetracycline in similar regions of tumours as a result of polypeptide complex linking or calcium metabolism.

Of particular interest was the absence of detectable uptake by 90–95 per cent of the cells of the three mouse ascites tumours investigated, even though these cells had been bathed in relatively high concentrations of fluorescent proteins for 24 hours or more. This result was identical with that obtained with two of these ascites tumours maintained *in vitro* (Easty *et al.*, 1964). The protein uptake by 5–10 per cent of the ascites cells has been discussed in detail (Easty *et al.*, 1964), and, excluding the absorption of fluorescent proteins by dead cells, three obvious possibilities were mentioned. Some of the cells which took up the proteins may have been viable tumour cells which differ strikingly from the majority in their capacity to pinocytose or phagocytose. Some may have been phagocytic cells of normal origin, including mesothelial cells which had become detached from the peritoneal body wall. Lastly, some may have been damaged tumour cells which had an increased capacity for pinocytosis (Thomason and Schofield, 1961). Similar observations have been made by Platt (1961), who found that squamous epithelial cells are stimulated to phagocytose under the influence of local trauma.

Previous investigations into the uptake of intact proteins by tumour cells *in vitro* have yielded conflicting results. Babson and Winnick (1954) found that whereas the uptake of radioactive amino acid by tumours could be inhibited by flooding with the nonradioactive isotope, the uptake of plasma protein labelled with that radioactive amino acid could not, which they interpreted as evidence for the uptake of intact protein by the tumour cells. Busch *et al.* (1961) also using proteins labelled with radioactive isotopes found that the specific activity of the protein within the tumour was higher than that found for the liver and other normal tissues studied. Campbell and Stone (1957) carried out very similar experiments on rats bearing azo-dye induced hepatomas, and obtained results suggesting that the protein was broken down into free amino-acids before being taken up by the tumour cells. It is difficult to see how allowance could have been made, using these techniques, for the uptake by normal macrophages and fibrous components which was frequently encountered in the work reported here. During homogenization of the tumour, desorption of intact labelled proteins from the tumour stroma could occur. The binding of the labelled proteins by fibres was not solely dependent on the presence of the label, since Mancini *et al.* (1962) obtained identical results using both the directly labelled proteins and fluorescent antibodies.

Cytological studies of protein uptake by tumour cells have been carried out by Cohen Beiser and Hsu (1961), using immuno-histochemical techniques. They observed the uptake of injected proteins by scattered cells of azo-dye induced hepatomas, but pointed out that the incorporation may have been related to cell damage incidental to the process of carcinogenesis. Ludford (1929, 1932) showed that the incorporation of injected trypan blue within the tumours of animals was confined to cells of the reticuloendothelial system, mainly localized at the tumour periphery. Ghose, Nairn and Fothergill (1962) obtained results which conflict with those described in this report. They observed in mouse ascites tumour cells and in the cells of solid tumours " a substantial amount of protein conjugate ". These conflicting results may be related to experimental variables as yet unknown.

It must be emphasized that the results described in this communication do not show that uptake of macromolecules by the majority of viable tumour cells cannot occur. The sensitivity of the technique is limited. What does emerge, however, is that reticuloendothelial cells within the tumour and normal tissues incorporate the injected proteins very much more rapidly than the tumour cells.

There have been suggestions, e.g. Starbuck and Busch (1962), that the preferential uptake by tumour cells of macromolecules carrying toxic agents might be of therapeutic value. If the specificity of action was dependent solely on the quantities taken into the cells, then from this investigation it would appear that sacrifice of the reticulo-endothelial system would probably precede death of the tumour cells. This objection would not apply to attempts to replace " deleted " macromolecules such as enzymes by means of reagents which were not toxic. However, little is known of the ultimate fate of macromolecules taken into the cells within vacuoles by pinocytosis or phagocytosis, and electron microscope studies (Ryser, Caufield and Aub, 1962 ; Easton, Goldberg and Green, 1962) have not, so far, yielded convincing evidence of the escape of intact proteins from the vacuoles into the cytoplasm.

The use of homologous, as opposed to heterologous proteins may well affect the uptake of proteins by normal and tumour tissues. For example, Nelson and Buras (1963) have found that homologous and heterologous red blood cells are phagocytozed at different rates ; the spleen primarily taking up homologous red cells and the liver primarily taking up heterologous red cells. Similarly, changes in the physiological condition of the host can have profound effects on the uptake of macromolecules by the cells of some tissues. Respiratory hypoxia, for example, induces active pinocytosis in rat liver parenchyma cells (Oudea, 1963). The possession by the injected macromolecules of properties such as enzymatic activity (Potter *et al.*, 1960) or antibody specificity (Pressman and Keighley, 1948) can also effect the distribution of macromolecules within tissues. These, and other factors need investigation in any attempts to achieve maximum uptake of macromolecules by tumour cells and minimum uptake by normal cells.

SUMMARY

1. The distribution of injected fluorescent-labelled diphtheria toxoid and bovine plasma albumin in some of the normal tissues and the tumours of tumour-bearing animals has been investigated. Twelve different tumours were examined.

2. Within the normal tissues examined, uptake of fluourescent proteins was chiefly confined to cells of the reticuloendothelial system and proximal tubule cells of the kidney. No uptake by the great majority of the tumour cells was detected.

3. Relatively large quantities of fluorescent protein were frequently observed within the tumours, but fluorescence was localized within macrophages and on the fibres of the tumour stroma. In the absence of stroma the proteins penetrated between and outlined the tumour cells without being taken up by the cells in detectable quantities.

The author wishes to acknowledge the invaluable assistance and advice given by Dr. J. A. H. Wylie in the histological aspects of this work. He also wishes to thank Mr. E. Woollard for the sections and Mr. M. Docherty for the figures. This investigation has been supported by grants to the Chester Beatty Research Institute (Institute of Cancer Research : Royal Cancer Hospital) from the Medical Research Council, the British Empire Cancer Campaign for Research, the Tobacco Research Council and the National Cancer Institute of the National Institutes of Health, U.S. Public Health Service.

REFERENCES

Babson, A. L. and Winnick, T.—(1954) *Cancer Res.*, **14**, 606.
Busch, H., Fujiwara, E. and Firszt, D.—(1961) *Ibid.*, **21**, 371.
Campbell, P. N. and Stone, N. E.—(1957) *Biochem. J.*, **66**, 669.
Cohen, S., Beiser, S. and Hsu, K.—(1961) *Cancer Res.*, **21**, 1510.
Easton, J. M., Goldberg, B. and Green, H.—(1962) *J. Cell Biol.*, **12**, 437.
Easty, G. C., Yarnell, M. M. and Andrews, R. D.—(1964) *Brit. J. Cancer*, **18**, 354.
Ghose, T., Nairn, R. C. and Fothergill, J. E.—(1962) *Nature, Lond.*, **196**, 1108.
Gitlin, D., Landing, B. H. and Whipple, A.—(1953) *J. exp. Med.*, **97**, 163.
Goldacre, R. J. and Sylvén, B.—(1962) *Brit. J. Cancer*, **16**, 306.
Kruse, H. and McMaster, P. D.—(1949) *J. exp. Med.*, **90**, 425.
Ludford, R. J.—(1929) *Proc. roy. Soc.* B., **105**, 493.—(1932) *Sci. Rep. Cancer Res. Fd Lond.*, **10**, 169.
Mancini, R. E., Vilar, J. M., Dellacha, O. W., Davidson, C. J., Gomez, C. J. and Alvarez, B.—(1962) *J. Histochem. Cytochem.*, **10**, 194.
Masouredis, S. P.—(1960) *J. Immunol.*, **85**, 623.
Nelson, E. L. and Buras, N. S.—(1963) *Ibid.*, **90**, 412.
Oudea, P. R.—(1963) *Lab. Invest.*, **12**, 386.

PLATT, H.—(1961) *Nature, Lond.*, **190**, 1075.
POTTER, J. L., MCCLUSKEY, R. T., WEISSMANN, G. AND THOMAS, L.—(1960) *J. exp. Med.*, **112**, 1173.
PRESSMANN, D. AND KEIGHLEY, G.—(1948) *J. Immunol.*, **59**, 141.
RYSER, H., CAULFIELD, J. B. AND AUB, J. C.—(1962) *J. Cell Biol.*, **14**, 255.
SCHILLER, A. A., SCHAYER, R. W. AND HESS, E. L.—(1952) *J. gen. Physiol.*, **36**, 489.
STARBUCK, W. C. AND BUSCH, H.—(1962) *Cancer Res.*, **22**, 1206.
THOMASON, D. AND SCHOFIELD, R.—(1961) *Exp. Cell Res.*, **24**, 457.
VASSAR, P. S., SAUNDERS, A. B. AND CULLING, C. F.—(1960) *Arch. Path.*, **69**, 613.